# Small molecules targeting histone demethylase genes (KDMs) inhibit growth of temozolomide-resistant glioblastoma cells

**DOI:** 10.18632/oncotarget.16820

**Published:** 2017-04-04

**Authors:** Barbara Banelli, Antonio Daga, Alessandra Forlani, Giorgio Allemanni, Daniela Marubbi, Maria Pia Pistillo, Aldo Profumo, Massimo Romani

**Affiliations:** ^1^ Laboratory of Tumor Epigenetics, IRCCS AOU San Martino–IST, Genova, Italy; ^2^ Department of Health Sciences, University of Genova, Genova, Italy; ^3^ Laboratory of Regenerative Medicine, IRCCS AOU San Martino–IST, Genova, Italy; ^4^ Department of Experimental Medicine (DIMES), University of Genova, Genova, Italy; ^5^ Biopolymers and Proteomic Unit, IRCCS AOU San Martino–IST, Genova, Italy

**Keywords:** glioblastoma, drug resistance, epigenetics, temozolomide, histone demethylase

## Abstract

In glioblastoma several histone demethylase genes (KDM) are overexpressed compared to normal brain tissue and the development of Temozolomide (TMZ) resistance is accompanied by the transient further increased expression of *KDM5A* and other *KDMs* following a mechanism that we defined as “epigenetic resilience”. We hypothesized that targeting KDMs may kill the cells that survive the cytotoxic therapy.

We determined the effect of JIB 04 and CPI-455, two KDM inhibitors, on glioblastoma cells and found that both molecules are more effective against TMZ-resistant rather than native cells.

Because of its lower IC50, we focused on JIB 04 that targets KDM5A and other KDMs as well. We have shown that this molecule activates autophagic and apoptotic pathways, interferes with cell cycle progression, inhibits cell clonogenicity and dephosphorylates Akt thus inactivating a potent pro-survival pathway. We performed combination temozolomide/JIB 04 *in vitro* treatments showing that these two molecules, under certain conditions, have a strong synergic effect and we hypothesize that JIB 04 intercepts the cells that escape the G2 block exerted by TMZ. Finally we studied the permeability of JIB 04 across the blood-brain barrier and found that this molecule reaches bioactive concentration in the brain; furthermore a pilot *in vivo* experiment in an orthotopic GB xenograft model showed a trend toward longer survival in treated mice with an Hazard Ratio of 0.5.

In conclusion we propose that the combination between cytotoxic drugs and molecules acting on the epigenetic landscape may offer the opportunity to develop new therapies for this invariably lethal disease.

## INTRODUCTION

For many years chemotherapy has had only a marginal role in glioblastoma (GB) treatment because of the intrinsic resistance of this tumor to chemotherapy and of the limited permeability of the blood-brain barrier to drugs [1–4]. Adjuvant temozolomide (TMZ) treatment resulted in a limited but significant benefit for the patients particularly if the *MGMT* gene is inactivated by DNA methylation in the tumor [3–5]. Nevertheless GB rapidly recurs becoming refractory to other treatments. The constitutive and acquired drug resistance in GB likely reflects the cellular and molecular heterogeneity of this tumor and the presence of Glioma-Initiating Cells, a cell population with distinct phenotypic and molecular characteristics, diverse differentiation potential and unique properties of invasiveness and self-renewal that is considered responsible for therapeutic failure and tumor recurrence [1, 6].

The epigenetic inactivation of the DNA repair gene *MGMT* has a pivotal role in the constitutive resistance to TMZ, whereas its role in acquired resistance is controversial [1, 3, 5, 7–11].

Utilizing an *in vitro* model of inducible drug resistance [12] we have shown that GB primary cultures enriched in cancer stem cells respond to the acute TMZ treatment by developing transient and reversible resistance through a mechanism that we have defined as “epigenetic-resilience” to describe the plasticity of tumor cells in response to “offending” stimuli [12]. We have hypothesized that the early, reversible, response is largely epigenetic and that is followed by further alterations that render GB cells irreversibly resistant to TMZ. In glioblastoma, several histone demethylase genes (*KDMs*) are overexpressed in comparison to normal brain cells and their expression is transiently further increased during the acquisition of TMZ resistance [12]. KDMs function as “partners” of oncogenes and are believed to have a central role in maintaining normal cell functions as well as in cancer cells biology and are considered potential targets in adult and pediatric glioblastoma [13–19] In particular, the overexpression of *KDM5A* was found in “Drug Tolerant Persister” cells (DTP), a subpopulation of tumor cells that give rise to expanded populations of drug resistant cells [20] including TMZ- resistant GB cells [12].

In agreement with the hypothesis that *KDM5A* is a driver of drug resistance in GB, we showed that the plasmid-mediated overexpression or RNAi-mediated silencing of *KDM5A* mimics TMZ resistance or sensitivity respectively [12]. KDM5A-mediated drug resistance likely is a mechanism common to different tumors since it has been described also in lung, prostate and breast cancer established cell lines [20–22]. On the other hand *KDM1* [14], and more recently *KDM6A/B* [13] were found to have a role in glioblastoma and *KDM1*, *KDM4A*, *KDM5A* and *KDM5B* are transiently overexpressed in GB cells that have acquired TMZ resistance [12].

For long time, selective KDM inhibitors (KDMi) have been available only for KDM1 [23]. Recently two KDMi, CPI-455 and YUCA1, were identified and found to inhibit the entire KDM5 family (CPI-455) or KDM5A and at a lesser extent KDM5C and to prevent the growth of drug-tolerant cells [24, 25].

Given the potential involvement of many *KDM* genes in GB, their targeting could be performed utilizing a cocktail of selective KDMi; however molecules possessing multiple specificities might be equally valuable.

JIB 04 is small molecule inhibiting the activity of the Jumonji family of KDMs [26] and, when tested on purified proteins, exerts its maximal inhibitory activity against KDM5A (IC50: 230 nM) and has, as secondary targets, KDM4D/4B/4A/6B/4C (IC50: 340–1100 nM). Beside KDM1, KDM4A and KDM5A/5B are up-regulated in TMZ-resistant GB cells [12], KDM4B is up-regulated in response to irradiation [27, 28] and KDM6B was identified as a possible therapeutic target in the childhood Diffuse Intrinsic Pontine Glioma (DIPG) [17]. JB 04 diminishes the growth rate of breast and lung cancer continuous cell lines *in vitro* and prolongs survival of mice with subcutaneous tumor xenografts [26]. As such, JIB 04 appears as a potential candidate for experimental therapies in GB.

Along this line, taking advantage of the multiple specificities of JIB 04, we have studied the activity of this molecule and of the KDM5-specific inhibitor CPI-455 against native and TMZ-resistant GB cells alone and in combination with TMZ as a preclinical step toward the development of combination therapies targeting the GB cells that escape the first line of therapy.

## RESULTS AND DISCUSSION

### JIB 04 and CPI-455 preferentially inhibit proliferation of TMZ-resistant GB cells

**Figure 1:**
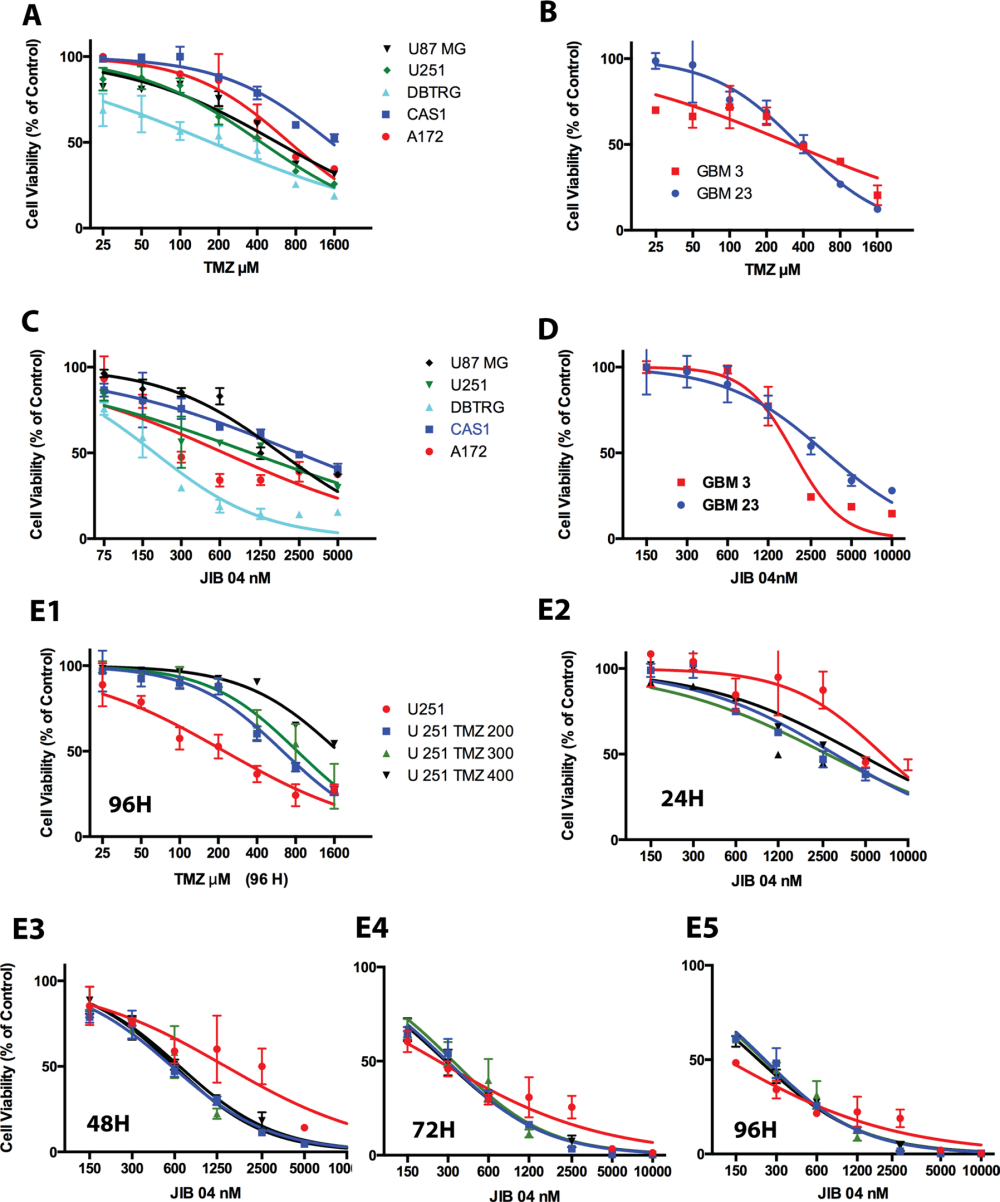
Effect of TMZ and JIB 04 on native GB primary cultures and cell lines (Panel **A**) Activity of TMZ on a panel of GB cell lines measured by MTS. Each point represents the mean value of three replicates. Treatment time: 72 hours. IC50 values were: A172 731μM; CAS1 1544 μM; DBTRG 175 μM; U87 MG 573 μM; U251 431 μM. (Panel **B**) Activity of TMZ on stem-enriched GB cells primary cultures measured by MTS. Treatment time: 72 hours. Each point represents the mean value of three replicates. IC50 values were: GBM3 324 μM; GBM 23 356 μM. The statistical significance of the effect of TMZ on cell viability (Panels A and B) is reported in Supplementary Table 1A. (Panel **C**) MTS analysis of GB cell lines exposed for 48 hours to JIB 04. Each point represents the mean value of three replicates. IC50 values were: A172 647 nM; CAS1 2400 nM; DBTRG 186 nM; U251 1047 nM; U87 MG 1784 nM. (Panel **D**) MTS analysis of patient's-derived GB primary culture exposed for 96 hours to JIB 04. Each point represents the mean value of three replicates. IC50 values: GBM 3 1860 nM, GBM 23 3200 nM. The statistical significance of the effect of JIB 04 on cell viability (Panels C and D) is reported in Supplementary Table 1B. (Panels **E1**–**E5**) RealTime-Glo MT analysis of U251 cells exposed to TMZ or JIB 04. U251 TMZ 200, TMZ 300 and TMZ 400 are U251 cells made resistant to TMZ after growth in 200, 300 or 400 μM TMZ. Each datapoint represents the average of three replicates and two separate experiments. IC50 values and significance of the observed differences is reported in Supplementary Figure 1, Panels 1B and 1C.

GB cells are intrinsically resistant to radio and chemotherapy. Nevertheless, resistance to TMZ is widely variable, and depends largely but not exclusively from the methylation of the *MGMT* gene [3, 5]. By MTS, we have evaluated TMZ sensitivity in a panel of GB cell lines and patients’-derived stem-enriched GB cells and found IC50 values comprised between 174 and 1500 μM for both cell types (Figure 1, Panels 1A and 1B, unpublished results, Supplementary Table 1A).

Having previously shown that GB cells overexpress several *KDM* genes and that the inhibition of *KDM5A* by shRNA sensitized TMZ-resistant GB cells to TMZ [12], we wanted to determine if the inhibition of the KDM5A enzyme activity had a similar effect. Accordingly we tested the activity on GB cells of two recently identified molecules possessing inhibitory activity on KDM: JIB 04 is a multi KDM inhibitor having its maximal activity against KDM5A [26] and CPI-455 is a molecule that selectively inhibits the KDM5 family [24].

As shown in Figure 1, Panels 1C and 1D, in Supplementary Figure 1, Panel 1A and in Supplementary Table 1B and 1C, the multispecific KDM inhibitor JIB 04 significantly inhibits the proliferation of GB cell lines and stem-enriched cultures. In general, the cells presenting higher TMZ resistance were also those less sensible to the effect of JIB 04 and this finding raised the possibility that TMZ resistance and JIB 04 sensibility could be interlinked.

**Figure 2:**
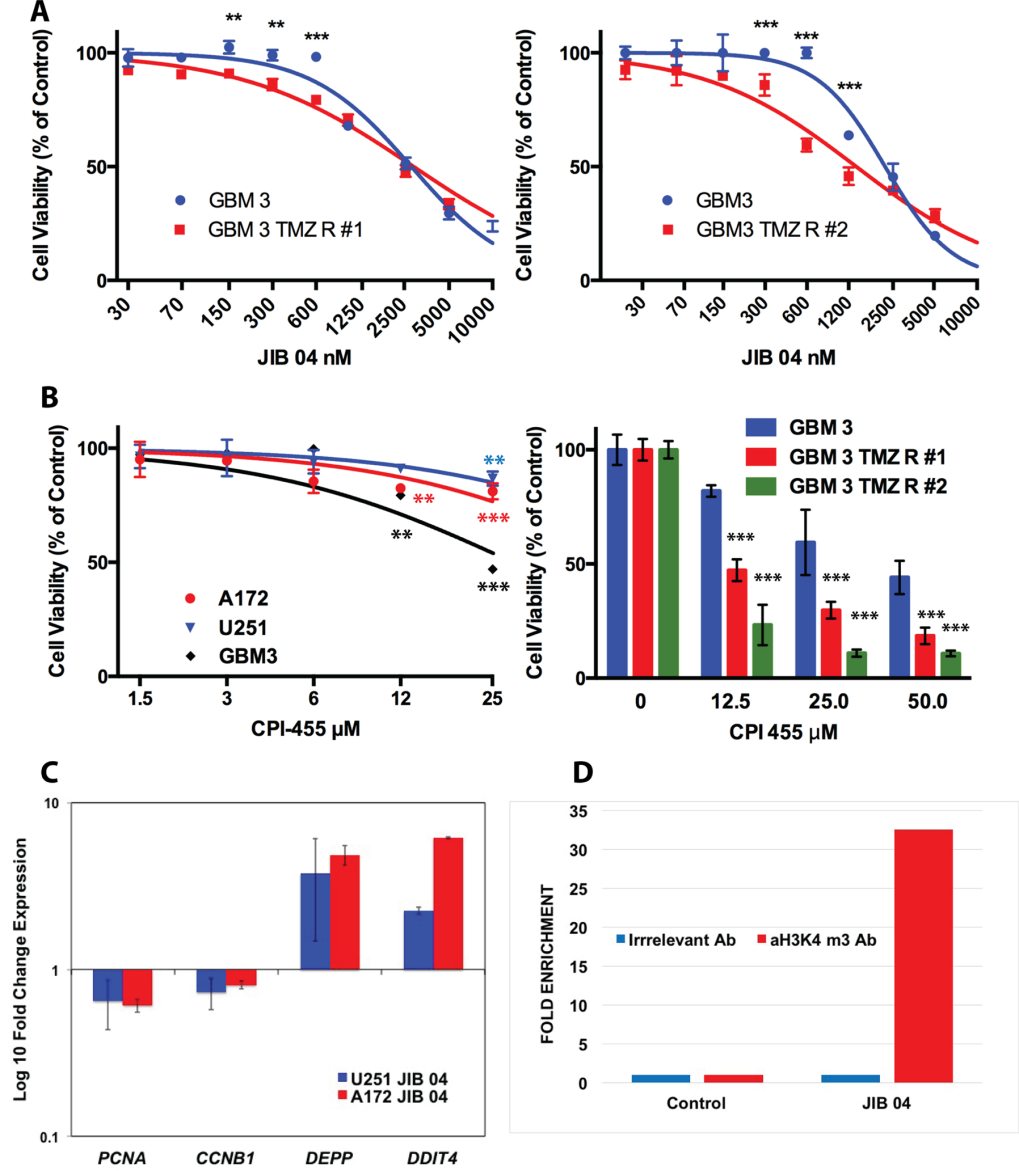
Effect of JIB 04 and CPI 455 on TMZ resistant GB cell lines and primary cultures and effect of JIB 04 on gene expression and H3 methylation (Panel **A**) Effect of JIB 04 on two TMZ resistant derivatives of the GBM3 stem-enriched culture obtained independently at a distance of one year. Treatment time 96 hours. Each datapoint represents the mean of triplicate assays. Significance of the difference of TMZ resistance between native and TMZ R cells was determined by ANOVA with the Bonferroni's correction for multiple comparisons and is indicated for each point (*P = 0.05; **P = 0.01; ***P = 0.001). (Panel **B** **left**): sensitivity of GB cells to the selective KDM5 inhibitor CPI 455 evaluated by MTS. Treatment time 120 hours. The significance of the difference between treated and control cells was determined by ANOVA as described for Panel A. (Panel **B right**): sensitivity of two independent TMZ resistant derivatives of the GBM3 stem-enriched culture to CPI 455 evaluated by MTS. Treatment time 120 hours. The significance of the difference between TMZ-resistant and native cells treated with the same concentration of CPI 455 was determined by ANOVA as described for Panel A. (Panel **C**) Expression of *CCNB1*, *PCNA*, *DEPP* and *DDIT4* genes in A172 and U251 cells exposed to JIB 04 for 4 hours normalized against untreated cells (fold-change = 1.0). (Panel **D**) Chromatin immunoprecipitation of A172 cells exposed to JIB 04 for 4 hours with an antibody against H3K4 me3 and with an irrelevant antibody. The immunoprecipitated was then amplified by qPCR with a set of primers for the promoter region of DEPP and the results show a > 30 fold enrichment for H3K4me3 over the irrelevant antibody only in the JIB 04 treated cells.

We then generated three U251 cell lines resistant to 200, 300 and 400 μM TMZ (Figure 1 Panel E1 and Supplementary Figure 1, Panel 1C) and we treated them with JIB 04 for 24, 48, 72 and 96 hours. As Shown in Figure 1, Panels E2–E5 and in Supplementary Figure 1, Panels 1B and 1C, after 24 hours of treatment the IC50 values of TMZ resistant cells were significantly lower than those of native cells. After 48 hours the IC50 values of the resistant cells was approximately half of that of native cells although this difference did not reach statistical significance. At 72 and 96 hours TMZ resistant and native cells were equally sensitive to JIB 04. A similar effect, although less evident, was seen also in two distinct TMZ-resistant patients’ derived, stem enriched GBM3 cells generated from the same parental culture at the distance of one year each from the other (Figure 2, Panels A1 and A2).

Overall these results demonstrate that the inhibition of KDM activity reduces the proliferation of glioma cells and that TMZ resistant cells are, up to a certain extent, more sensitive to JIB 04 than native cells likely because these cells, overexpressing KDM genes, are more efficiently targeted by KDM inhibitors.

To test the effect of JIB 04 on non- or slow-proliferating cells, we cultured GB cells grown in stem-permissive conditions or induced to differentiate by the addition of serum to the medium. Under differentiated conditions the cells reduce their proliferative activity and eventually stop dividing while undergoing morphological changes (Supplementary Figure 2, Panel 2A). Stem and differentiating primary GB cultures present striking differences in JIB 04 sensitivity and, as shown in Supplementary Figure 2, Panel 2B, the differentiating cells that proliferate slowly or do not proliferate at all are resistant to JIB 04 over a wide range of concentrations compared to their stem-enriched, proliferating, counterpart.

JIB 04 exerts its maximal inhibitory activity against KDM5A. However, since this molecule is active also against other KDMs overexpressed in glioma cells, it was impossible to unequivocally attribute its effect on cell survival to the inhibition of a specific KDM and particularly to KDM5A that we have hypothesized to be a mediator of drug resistance in GB cells [12]. To directly address the role of the KDM5 family in the survival of TMZ-resistant GB cells we have utilized the selective KDM5 inhibitor CPI 455 [24]. As shown in Figure 2, Panel 2B, CPI 455 had a modest but significant effect on cell viability of native GB cells only at high concentration. Conversely, the activity of CPI 455 against independently derived TMZ resistant cell was significantly stronger than that exerted on the parental cells (Figure 2, Panel 2C). Overall this result demonstrates that the inhibition of the KDM5 enzyme activity reduces the proliferation of TMZ resistant GB cells and fully agrees with our previous results showing that silencing *KDM5A* sensitizes the cells to TMZ. Moreover, the overexpression of *KDM* genes in TMZ resistant GB cells, increasing KDM enzymatic activity, render these cells preferential targets for these molecules.

Although both JIB 04 and CPI 455 are active against TMZ resistant GBM cells we focused our further study on JIB 04 because its IC50 is much lower than that of CPI 455 making easier, in principle, to reach clinically relevant concentration for *in vivo* studies.

### JIB 04 treatment modulates the expression of genes involved in the control of cancer cell growth and leads to hypermethylation of H3K4

Previous mRNA profiling studies conducted on the non-small cell lung cancer cell line H358 showed that JIB 04, within 4 hours of treatment, upregulated approximately 100 genes including many negative regulators of proliferation and genes responsive to DNA damage or to oxidative stress and downmodulated a set of 20 genes that includes several pro-growth genes [26]. We have examined, in GB cell lines A172 and U251, the effect of JIB 04 on the transcription of two proliferation genes (*CCNB1* and *PCNA*), and of two other genes that, at large, could be functionally considered as tumor suppressors (*DEPP* and *DDIT4*). The stress-responsive *DEPP* gene (c10orf10) leads to the activation of the autophagy pathway [29] whereas *DDIT4* is a gene responsive to hypoxia and DNA damage and activates p53-mediated apoptosis [30].

As shown in Figure 2, Panel C, short (4H) treatment of A172 and U251 downmodulated *CCNB1* and *PCNA* and strongly upmodulated *DEPP* and *DDIT4*. To explore the mechanism of JIB 04 induced overexpression of *DEPP*, we performed chromatin immunoprecipitation with an antibody against trimethylated H3K4 followed by real time amplification and, consistently with the inhibitory activity of JIB 04 on KDM5A, we found that the chromatin region upstream the transcription start site of *DEPP* was highly enriched in H3 trimethylated at K4 (Figure 2, Panel 2D).

### JIB 04 activates the autophagy and apoptotic pathways and inactivates PI3K

Having determined that JIB 04 is a potent inhibitor of the proliferation of GB cells either native or resistant to TMZ, we explored some of the mechanisms that potentially could take part in the antitumor activity of this molecule.

**Figure 3:**
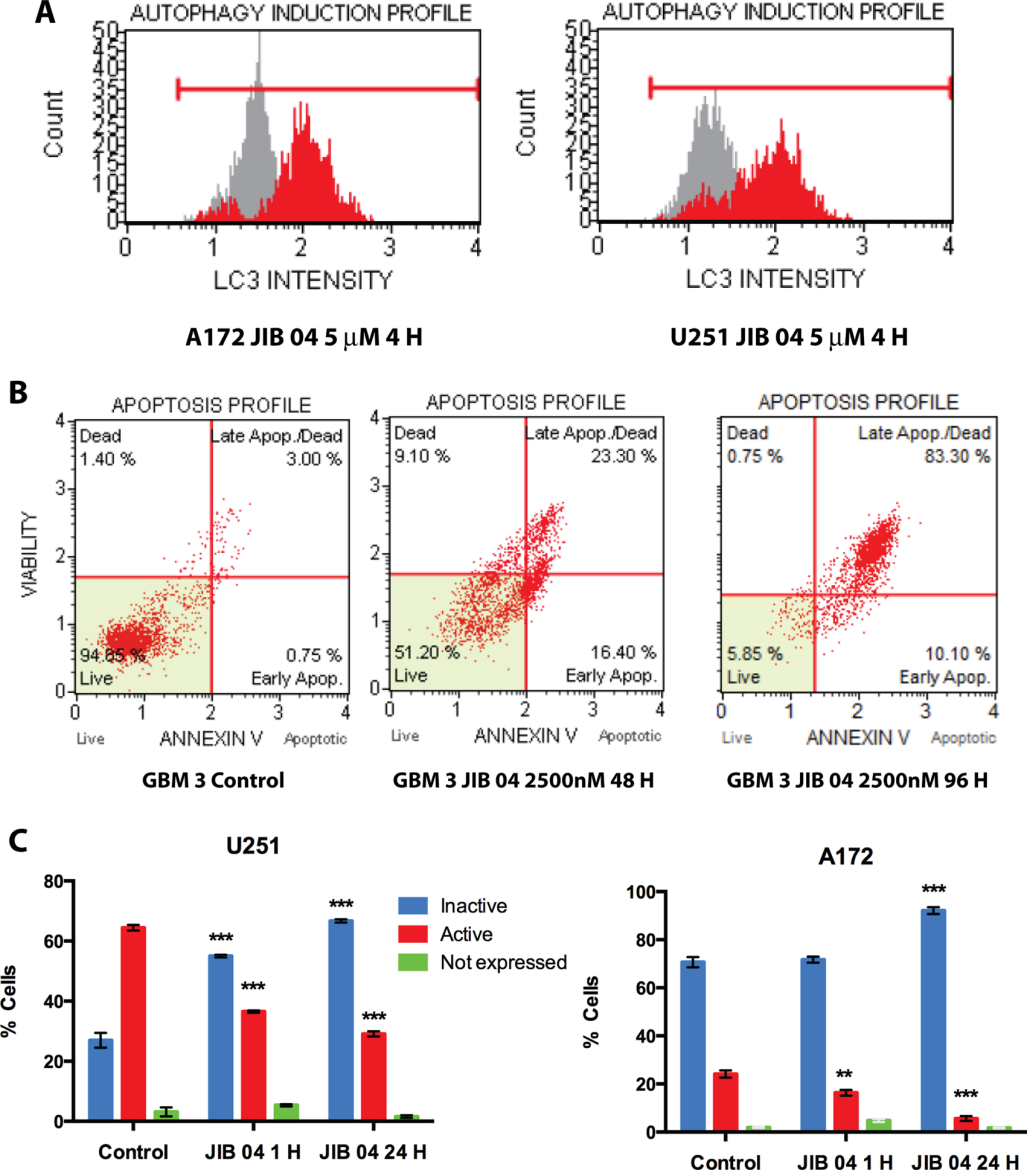
Functional effects of JIB 04 treatment on GB cells (Panel **A**) Cytofluorimetric analysis of LC3 translocation into autophagosomes upon treatment of GB A172 and U251 cells with JIB 04 for 4 hours. Red and gray profiles represent the test and control sample, respectively. Autophagy induction is the ratio between the mean autophagy intensity of the treated sample and that of the untreated, control sample. (Panel **B**) scatterogram of Annexin V staining of GBM 3 cells upon treatment with JIB 04 (2500 nM) for 48 and 96 hours. (Panel **C**) Flow-cytometry analysis of Akt phosphorylation after 1 and 24 hours of treatment with JIB04 of U251 and A172 cells. These plots show the presence of significant decrease in the phosphorylation of Akt indicating inactivation of the PI3K pathway. Each datapoint represents the mean value of two experiments. The significance of the differences respect the untreated cells was calculated determined by ANOVA with the Bonferroni's correction for multiple comparisons and is indicated for each point (*P = 0.05; **P = 0.01; ***P = 0.001).

To determine if the overexpression of *DEPP* in response to JIB 04 could activate the autophagy pathway we measured by flow cytometry the extent of LC3 translocation into autophagosomes [31] in A172 and U251 cells treated with JIB 04 for 4 hours. As show in Figure 3, Panel 3A, the exposure to JIB 04 for 4 hours, induced autophagy by 4.0 and 5.1 folds (red profile) over untreated cells (grey profile) in A172 and U251 cells respectively.

Longer (48–96 hours) exposure of the cells to JIB 04 resulted in the time-dependent significant strong induction of apoptosis under a wide range of molecule concentrations in both primary and established cultures as well as in TMZ resistant GB cells (Figure 3, Panel 3B, Supplementary Figure 3, Panels 3A–3C, and Supplementary Table 2).

The activation of the PI3K pathway (phosphatidylinositol 3-kinase (PI3K)/Akt/rapamycin-sensitive mTOR-complex (mTOR) pathway) is one of the most potent pro-survival pathways whose inappropriate activation is a frequent finding in human tumors. Targeting the PI3K pathway is considered one of the most promising avenues for cancer treatment [32]. In glioblastoma the levels of Phospho-PI3K, Phospho-Akt and Phospho-p70^s6k^, indicative of the PI3K activation, were associated with reduced apoptosis and shorter survival time of the patients [33]. Importantly, the activation of this pathway was put in relation with resistance to TMZ and radiotherapy in GB [33, 34]. Consequently, targeting the PI3K pathway in GB patients is being actively exploited as a potential innovative therapy for glioblastoma [35].

We have studied by cytometry the effect of JIB 04 on the phosphorylation of Akt at Ser473, an activating modification of Akt. We have analyzed a panel of primary and established GB cells for Akt activation and found that the baseline level of Akt phosphorylation is widely variable (from more than 80% to less than 5% of positive cells, data not shown). In U251 the majority of the cells carries phosphorylated Akt (Figure 3, Panel 3C, left) but the treatment with 1 μM JIB 04 for 1 hour was sufficient to significantly decrease the extent of Akt phosphorylation and to increase the fraction of cells bearing inactivated Akt. After 24 hours the effect of JIB 04 on Akt phosphorylation was even more evident and in the majority of the cells Akt was inactivated. In A172 Akt is constitutively inactive in more than 70% of the cells (Figure 3, Panel 3C, right ). Nevertheless, the treatment with JIB 04 resulted in the significant decrease of the number of cells bearing the activated form of Akt (from 23.1 to 6.5 %) with the consequent increase of those with the inactive form. After 24 hours of treatment in 91.1 % of the cells Akt was inactivated. In U251TMZ R cells, phosphorylation of AKT was higher than that of the native counterpart (77.5 vs 63.9) and the decrease of Akt phosphorylation upon JIB 04 treatment was significant but much less evident than that of the native cells after 1 hour of treatment. However, after 24 hours the decrease of phosphorylation became much more evident and highly significant (Supplementary Figure 2, Panel 3D). Overall these results confirm the role of the PI3K pathway in drug resistance and demonstrate that the KDM inhibitor JIB 04 can interfere with multiple pathways involved in the control of tumor cell growth in native and TMZ resistant cells.

### A brief treatment of GB cells with JIB 04 is sufficient to strongly reduce clonogenicity

**Figure 4:**
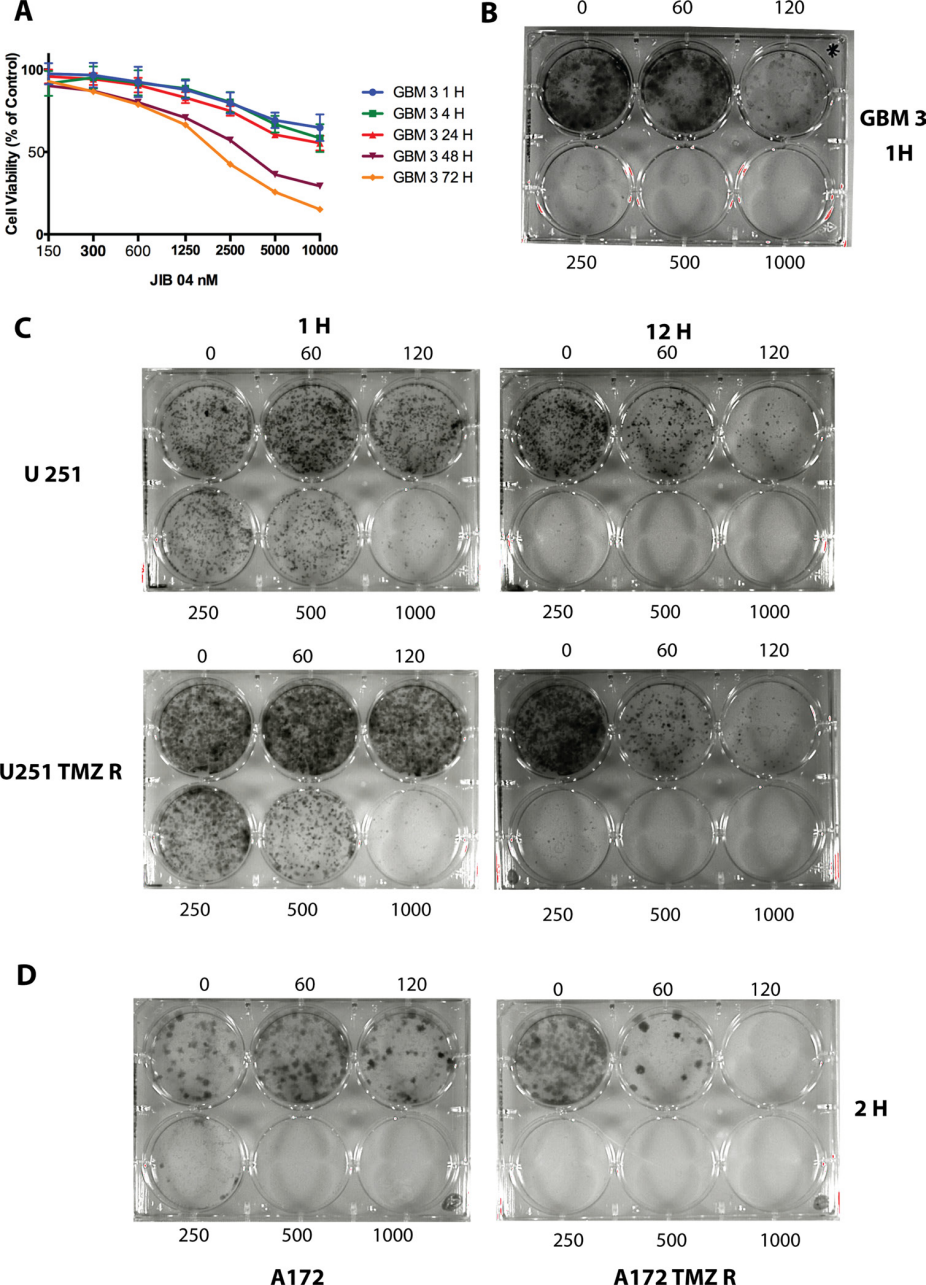
JIB 04 is a fast-acting molecule that inhibits clonogenicity (Panel **A**) Kinetic of JIB 04 activity on GBM3 cells at 1, 4, 24, 48 and 72 hours of treatment showing that the reduction of cell vitality becomes evident after 1 hour of treatment at the highest molecule concentrations. The significance of the effect of JIB 04 respect untreated cells at each time point for each dose of the molecule is reported in Supplementary Table 3. (Panel **B**) Clonogenicity of the GBM3 stem-enriched culture after 1 hour treatment with JIB 04. Range of concentrations: 60–1000 nM). (Panel **C**) Clonogenicity of U251 (upper part of the panel) and of U251 TMZ R (lower part) GB cells after 1 and 12 hours treatment with JIB 04. Range of concentrations 60–1000 nM. (Panel **D**) Clonogenicity of A172 (left) and of A172 TMZ R (right) GB cells after 2 hours treatment with JIB 04. Range of concentrations 60–1000 nM.

The dephosphorylation of Akt after a 1-hour exposure of the cells to JIB 04 prompted us to investigate the biological consequences of this brief treatment. We initially measured cell viability by Real-Time MT (see Materials and Methods) after treatment of the primary GBM3 cell culture with JIB 04. As shown in Figure 4, Panel 4A and in Supplementary Table 3, the significant reduction of cell viability was time and dose-dependent and was detectable, at high JIB 04 concentrations, 1 hour after the beginning of the treatment. The extent of reduction slightly increased after 4 and 24 hours reaching its maximum level after 72 hours. We initially tested the effect of JIB 04 on clonogenicity after treatments of 24 and 48 hours and found that, under these conditions, no colonies could be detected in native and TMZ-resistant GB cells at the concentrations tested (600 and 1200 nM, data not shown). The effect of JIB 04 on clonogenicity is evident even at low concentration and short exposure time. Indeed, the dramatic reduction of the clonogenic property of the cells can be observed, in primary and established cell lines, even after 1 hour of treatment (Figure 4, Panels 4B, 4C and 4D). Importantly, the potent inhibitory effect of JIB 04 on colony formation is not restricted to native cells and extends to the TMZ resistant derivatives with similar kinetics (Figure 4, Panels 4C and 4D).

### JIB 04 cooperates with TMZ in killing GB cells

TMZ, along with surgery and radiotherapy, is part of the standard care for glioblastoma patients. Unfortunately the clinical efficacy of TMZ is diminished by the therapy-driven chemoresistance and recurring tumors are often hypermutated and resistant to further therapies [36]. We have shown that the early stages of TMZ resistance are epigenetically-driven and partially reversible and we reasoned that hitting the cells that escape from TMZ treatment before acquiring mutations leading to irreversible drug resistance could offer new opportunities for GB treatment. In this respect JIB 04 is a molecule that, in principle, could potentiate the effect of TMZ since inactivates several KDMs including the product of the *KDM5A* gene whose overexpression or silencing mimics TMZ resistance or sensitivity, respectively [12].

**Figure 5:**
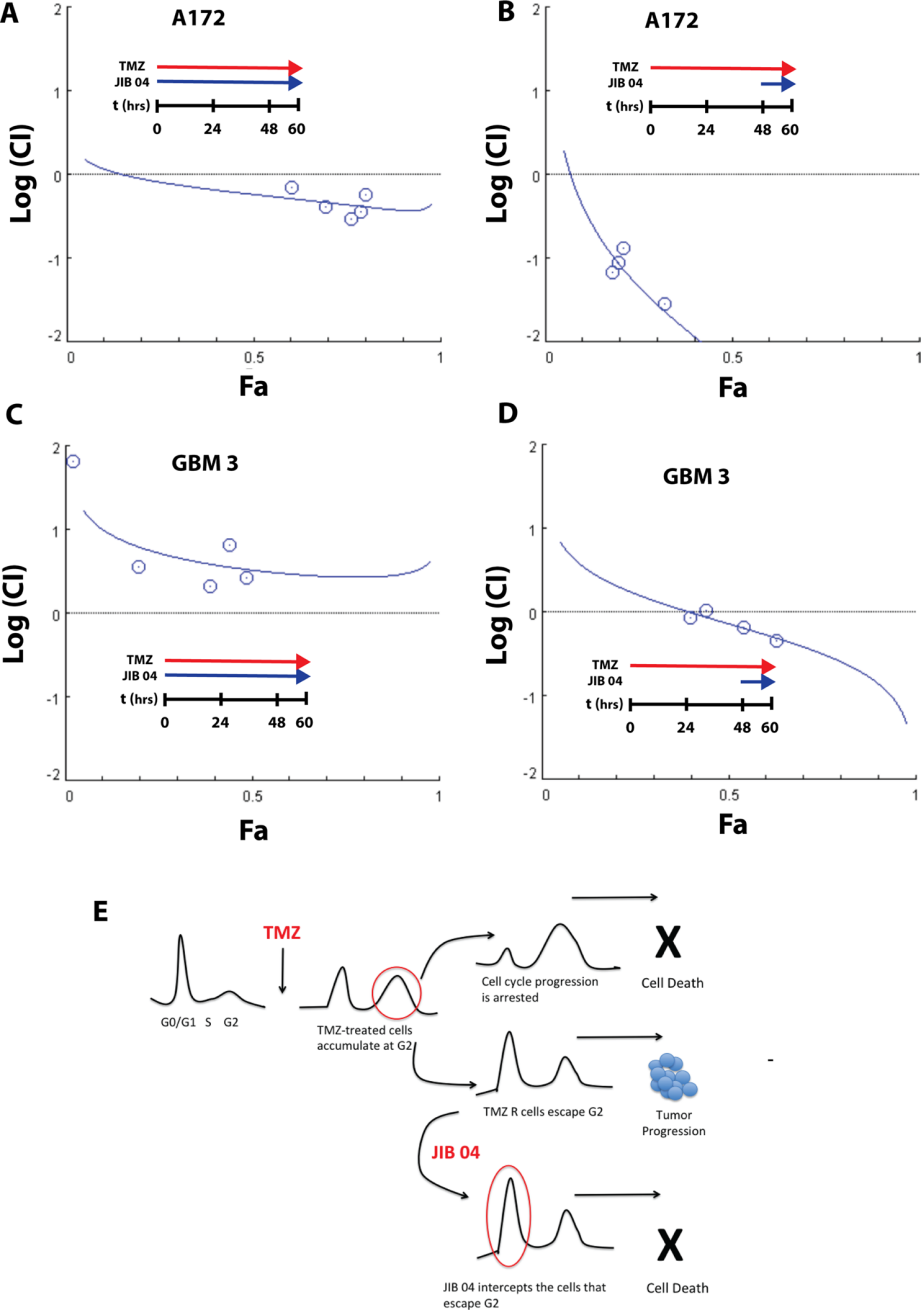
Synergy between JIB 04 and TMZ (Panel **A**) Plot of the log(CI) index vs. the effect (Fa) of JIB 04 treatment of A172 cells. The effect considered was the induction of apoptosis measured by Annexin V staining. Each data point represents the mean of duplicate analysis. Cells were incubated for 60 hours with a constant ratio of the two molecules (JIB 04 = 1; TMZ = 500). The plot of the CI indicates a moderate synergy between the two molecules. (Panel **B**) same as Panel A except that the cells were incubated for 48 hours with TMZ alone, and for 12 hours with TMZ and JIB 04. The ratio between JIB 04 and TMZ was the same of Panel A. The plot of the CI indicates a very strong synergy between the two molecules. (Panel **C**) Plot of the log(CI) index vs the effect (Fa) of JIB 04 treatment on GBM 3 primary GB cultures. The effect considered was the cell vitality measured by MTS. Each data point represents the men of triplicate analysis. Cells were incubated for 60 hours with a constant ratio of the two molecules (JIB 04 = 1; TMZ = 500). The plot of the CI indicates a modest to moderate antagonism between the two molecules. (Panel **D**) same as Panel C except that the cells were incubated for 48 hours with TMZ alone, followed by 12 hours with TMZ and JIB 04. The ratio between JIB 04 and TMZ was the same of Panel C. The plot of the CI indicates an additive effect at the lowest concentration and moderate synergy between the two molecules at higher concentrations. (Panel **E**) Working hypothesis on the mechanism of the combined effect of JIB 04 and TMZ. TMZ blocks the cell cycle at the G2 checkpoint. The cells that acquire TMZ resistance can pass G2 and resume proliferation. JIB 04 target these cycling cells cooperating with TMZ (see also Supplementary Figure 4 for the effect of JIB 04 on cell cycle).

We initially performed combination experiments cultivating A172 and GBM 3 cells simultaneously with TMZ and JIB 04 and monitoring the effects of the two molecules by MTS and induction of apoptosis. The dose-effect relationships of the drug combination were evaluated utilizing the mass-action law and the median-effect equations to calculate the Combination Index (CI) as measure of the interaction between TMZ and JIB 04 as described by Chou and Talalay [37]. According to this equation, a CI < 1 is indicative of synergy between two drugs whereas CI = 1 and CI > 1 indicate additive or antagonistic effects, respectively. As shown in Figure 5, Panel 5A, the combination of TMZ and JIB 04, showed a weak synergism between the two molecules (CI: 0.30–0.71). The synergy between the two molecules became very strong when the cells were exposed to TMZ only for 48 hours followed by further 12 hours of JIB 04 treatment (CI: 0.02–0.1) (Figure 5, Panel 5B). A conflicting result was initially obtained when we tested the TMZ-JIB 04 combination on the primary stem-enriched GBM 3 culture. As shown in Figure 5, Panel 5C, the two molecules showed a moderate to strong antagonist effect if utilized together (CI: 2.1 -> 10). However, when the cells were exposed first to TMZ for 48 hours and then to JIB 04 for 12 hours, the combination showed, as in A172, a synergic effect that turned into additive only at the lowest drug concentration (CI: 0.44–1.06) (Figure 5, Panel 5D). This result likely reflects the cellular heterogeneity of primary GB cultures that mimic the molecular and cellular complexity of the tumor.

Although we do not yet have a mechanistic support to explain the synergy between JIB 04 and TMZ when administered sequentially, we hypothesize that, under our experimental conditions, this effect could be the result of the different interference of TMZ and JIB 04 on cell cycle. It is known that TMZ-treated GB cells accumulate at the G2 checkpoint. and we have shown that the cells that acquire TMZ resistance overcome the G2 checkpoint and continue to proliferate entering in G1 [12]. The cell cycle analysis of GB cells treated with JIB 04 for 48 hours, show the progressive dose-dependent accumulation in G0/G1 (Supplementary Figure 4). We hypothesize that JIB 04 intercepts the cells that escape G2 before they progress through the cell cycle and eventually acquire new mutations that renders them permanently drug resistant.

### *In vivo* analysis of the effect of JIB 04 in an orthotopic mouse model of GB

In spite of many clinical and experimental trials with novel drugs, no substantial improvement in survival was obtained over the standard protocol. Many anticancer agents strongly active *in vitro* against GBM cells cannot penetrate and diffuse the brain because the Blood Brain Barrier (BBB) impedes the delivery of clinically relevant concentrations of these drugs and the tight grid formed by glial cells strongly limits the diffusion of therapeutic molecules at the tumor site.

For the initial assessment of acute toxicity *in vivo* we utilized JIB 04 from different manufacturers. Although the effect on cell proliferation *in vitro* was essentially equivalent between JIB 04 from different Companies (Supplementary Figure 4, Panel 4B), we observed that adverse effects *in vivo* occurred between 80 and 110 mg/kg depending from the batch and from the manufacturer (data not shown).

**Figure 6:**
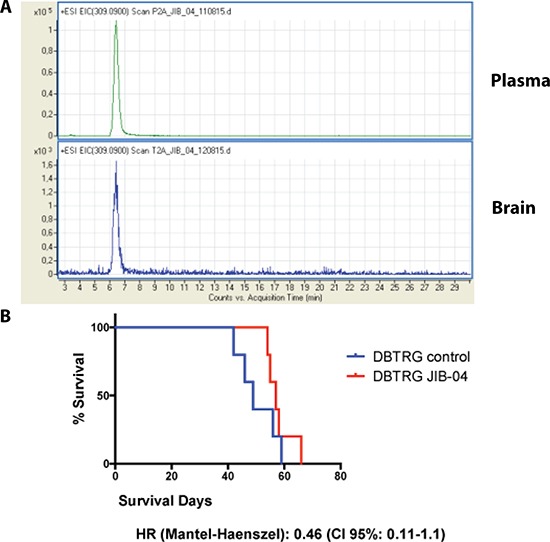
*In vivo* analysis of JIB 04 (Panel **A**) M Pharmacokinetics of JIB-04: extracted ion current of m/z 309.09 [M+H]^+^ in plasma and brain extracts of mice treated for 5 days with 60 mg/kg of JIB 04. The presence of an identical peak corresponding to JIB 04 (see Supplementary Figure 5) in the two extracts indicates the passage of the molecule through the blood brain barrier. (Panel **B**) Kaplan-Maier plots of 10 mice orthotopically xenografted with DBTRG cells treated with JIB 04 (N: 5) or with placebo (N: 5). The hazard ratio of the mice treated with JIB 04 is 0.5.

To determine if JIB 04 could pass through the BBB we developed a mass spectrometry protocol to unequivocally identify this molecule in tissue extracts (Supplementary Figure 5). We then performed a preliminary biodistribution analysis in 6 mice treated with 50 mg/kg of JIB 04 in a single i.p. administration. Under these conditions we observed a plasmatic peak at 30′ followed by the accumulation of the molecule in the liver and kidney. Mass spectrometry of whole brain extract showed detectable amount of JIB 04 indicative of the passage of the molecule through the BBB (data not shown). To determine if clinically relevant amount of JIB 04 could reach the brain, we treated daily for 5 days 9 mice with 60, 40 and 20 mg/kg of JIB 04, a schedule well tolerated by the mice. At the end of the treatment the mice were sacrificed and the concentration of JIB 04 was measured in the brain and in plasma. As shown in Figure 6, Panel 6A, JIB 04 was readily detectable in the brain of healthy mice with an intact BBB and at the dosage of 60 mg/kg the concentration in the brain reached approximately 200 nM.

In an earlier study it was demonstrated that JIB 04 inhibits cancer growth in mice xenografted with a non-small lung carcinoma cell line [26]. To assess the antitumor effect of JIB 04 in a stringent orthotopic context, we performed a pilot *in vivo* treatment in 5 mice xenografted in the brain with the GB cell line DBTRG and compared utilizing the product-limit method (Kaplan-Meier), the outcome of the treated mice with that of other 5 xenografted mice that received the vehicle alone. As shown in Figure 6, Panel 6B, the survival curves of treated and control mice were clearly distinct, but the difference did not reach statistical significance possibly because of the very limited number of mice included in this pilot study. Nevertheless, the Hazard Ratio of the JIB 04 treated mice/placebo was about 0.5, suggesting that indeed this molecule can interfere with the growth of glioblastoma cells *in vivo* in an orthotopic setting.

## MATERIALS AND METHODS

### Cell lines, GB CSC cultures and drugs

The human GB cell lines A172, U251, CAS1, DBTRG and U87 were obtained from the Biological Bank and Cell Factory of IRCCS AOU San Martino - IST (www.iclc.it) and were grown in DMEM supplemented with 10% FBS and 2 mM L-Glutamine. The authenticity of the cells was certified by the Biological Bank utilizing eight highly polymorphic STR loci plus amelogenin (Cell IDTM, Promega).

GB cultures derived from primary GB, were cultivated in stem-permissive, serum-free medium as previously described [49, 50]. Cell differentiation was carried out by shifting GBM CSCs in serum-containing medium (10% FBS). TMZ resistance was induced as previously described [12].

Temozolomide (TMZ) was acquired from Sigma-Aldrich (Milano, Italy), JIB 04 from Tocris (Bristol, UK) or Selleckchem (Munich, Germany) and CPI 455 was from Axon Medchem (Groningen, NL). TMZ and CPI 455 were dissolved in DMSO at 100 and JIB 04 was dissolved in DMSO at 10 mM. All chemicals were stored at −80°C and diluted in culture medium immediately before use.

### Cellular analyses

Cell proliferation and viability were measured by colorimetric MTS (One Solution Cell Proliferation Assay, Promega, Milano, Italy) or, when indicated, by continuous-read luminescent MT assay (RealTime-Glo MT Cell Viability Assay, Milano, Italy).

Cell cycle analysis, apoptosis (measured by annexin V staining), induction of autophagy measured by LC3 staining into autophagosomes and PI3K activation were determined utilizing a Muse Cell Analyzer and the dedicated kits (Millipore-Merck, Vimodrone MI, Italy).

Cell assays were performed in duplicate or triplicate and each experiment was repeated at least twice. Clonogenicity assays were performed as previously described [12].

### Quantitative real-time reverse transcription-PCR and Chromatin Immunoprecipitation (CHIP)

Real Time PCR analysis was carried out as described [12]. Primers for *CCNB1, PCNA* and *DEPP* were those utilized by Wang et al. [26], Primers for *DDIT4* were designed by Primer 3 (http://primer3.ut.ee/) and their sequence is: FW-GGTCACTGAGCAGCTCGAA; REV-CCTGGACAGCAGCAACAGT. Relative quantification of each target gene transcript was obtained using comparative Ct method. Reference genes (*ATP5b*, *SDHA1*, and *CYC1*) were selected using GeNorm (PrimerDesign Ltd, Southampton, UK). For each cDNA, the duplicate Ct values were averaged and normalized (geometric mean). The copy number was expressed relative to a calibrator sample using the 2−(ΔΔCt ± SD) method.

CHIP was performed utilizing the Epitect® ChIP One Day Kit (Qiagen, Milano, Italy) utilizing the technical conditions reccomended by the manufacturer. Immunoprecipitation was performed with an anti H3K4me3 Polyclonal Antibody (Epigentek, Farmingdale NY, USA). As irrelevant antibody we utilized a chicken alpha-GFP Antibody (Invitrogen – Thermo Fisher, Waltham, MA, USA). Evaluation of the immunoprecipitate was performed by qPCR utilizing the primers described in Ref. [26]

### Statistical analysis and drugs interaction

Statistical significance was determined by analysis of variance (ANOVA) and post-hoc analysis (Bonferroni correction for multiple comparisons). The accepted value of significance was 0.05. All statistical analyses were performed using Graph Pad Prism software (GraphPad Software, San Diego CA, USA).

Interaction between TMZ and JIB 04 was evaluated by testing the activity of TMZ and JIB 04 combined together at a constant ratio. The Combination Index (C.I.) that quantitatively express drug interaction, was calculated according to the Chou-Talalay equations utilizing the CompuSyn software (www.combosyn.com) [37].

### *In vivo* experiments and animal model

For *in vivo* experiments mice were housed in pathogenic-free conditions, and they were handled in agreement with guidelines conforming to current Italian regulations for the protection of animals used for scientific purposes (D.lvo 27/01/1992, no.116).

Procedures were approved by the Ethical Committee for Animal Experimentation of the National Institute of Cancer Research and by the Italian Ministry of Health (#751/2016-PR) and were performed according to the National Regulation on Animal Research Resources.

For the evaluation of JIB-04 toxicity and organ distribution, groups of 4 C57 BL6/J mice (8 weeks old; Charles River Laboratories, Lecco, Italy) were injected i.p. with singles scalar doses of the drug, up to 110 mg/kg in 10% DMSO 90% sesame oil. Blood samples (0,1 ml) were collected by retro-orbital bleeding at 30 min, 3 and 6 hours after drug administration. Following mice euthanasia after the last bleeding time point, brains were harvested weighted and homogenized in ice-cold water (5 ml/g tissue). For chronic administration, the same procedure was repeated after 5 days dosage at 5, 10 or 20 mg/kg/die.

Intracranial tumor inoculation was performed on adult NOD-SCID mice (6–8 weeks old; Charles River Laboratories, Lecco, Italy) as already described (PMID: 24443327). Briefly, animals were positioned into a stereotactic apparatus and injected into the left striatum with 3 μ l of cell suspension, containing 3e5 trypan blue-negative DBTRG cells. One week after tumor inoculation, mice were randomly assigned to treatment and control groups (5 mice/group). Treated mice received 80 mg/kg 3 times weekly for 3 weeks. Mice were monitored daily and sacrificed at the onset of neurological symptoms.

### Pharmacokinetic of JIB 04

For each sample 205 μl of acetonitrile were added to 45 μl of tissue homogenate. Samples were vortexed for 30 seconds, incubated at room temperature for 10 min and then centrifuged for 15 min. Finally, 200 μl of supernatant were collected, lyophilized and stored at −20°C until used. The same procedure was also applied for the analysis of the plasma samples. A JIB 04 calibration curve was prepared in brain homogenate. The concentration of the calibration curve ranged from 0.005 μM to 10 μM containing the following JIB 04 concentration: 0.005, 0.01, 0.05, 0.1, 0.5, 1, 5, 10 μM. Each point was assayed in duplicate. JIB 04 content was determined by high performance liquid chromatography (HPLC)/mass spectrometry (MS). The separation was carried out using an HPLC system consisting of a vacuum degasser, an autosampler, a capillary pump and a thermostated column compartment (Agilent series 1200, Agilent Technologies, Palo Alto, CA, USA). Briefly, the lyophilized samples were reconstituted with 60 μl of acetonitrile and sonicated for 5 minutes, then 40 μl of water were added to each tube and the samples were centrifuged for 15 min. Two microliters of sample were injected onto a 0.5 mm × 250 mm, 5 μm particle size, ZORBAX SB-C18 column (Agilent Technologies). Mobile phases A was 20 mM ammonium formate in acetonitrile–water-formic acid (60:40:0.1); mobile phase B was isopropanol-acetonitrile (80:20). The flow rate was 20 μl/min, and the elution was performed in this sequence: isocratic 100% A for 10 min, a linear gradient over the course of three min to 100% B, maintained at 100% B for 25 min and finally a linear gradient to 100% A in three min. The re-equilibration time in 100% A was 25 min. Although the JIB 04 elution takes place during the first isocratic step, a strong hydrophobic washing step is required to assure the complete elution of highly lipophilic molecules contained in the brain tissues. The eluent flow was directly sent to the electrospray (ESI) ion source of the 6210 Time of Flight mass spectrometer (Agilent Technology) to characterize the HPLC peaks. The following operation parameters were applied: capillary voltage: 3000 V; nebulizer pressure: 20 psig; drying gas: 5 L/min; gas temperature: 300°C; fragmentor voltage: 200 V; skimmer voltage: 60 V; octapole RF: 250 V. The instrument performed the internal mass calibration automatically, using a dual nebulizer electrospray source with an automated calibrant delivery system. The full-scan data, recorded using the Agilent's Mass Hunter software, were processed with Mass Hunter Qualitative Analysis (Agilent Technology). Mass spectra, ranging from 100-1000 m/z, were acquired in reflectron positive ion mode and the relative amount of JIB-04 was measured by extracted ion current (EIC) peak area (m/z 309.09 [M+H]^+^).

## CONCLUSIONS

In an attempt to improve the outcome of GB patients, many clinical trials were launched in the last years to test the efficacy of consolidated “biological drugs”, and of targeted and immunological therapies active against many other tumor types (reviewed in ref. [38]). Overall, the results of many phase I/II and III clinical trials were highly disappointing showing, at best, a very limited efficacy often with severe adverse effects [38–40]. The results of the ongoing phase I/II/III trials that include the blockers of “immune checkpoints” PD1 and/or CTLA-4 that have a proved efficacy in other tumors [41] and in preclinical GB models [42] appear promising, although not impressive [43, 44] .

In the recent years the recognition of the involvement of epigenetic mechanisms and epigenetic alterations in cancer heterogeneity and drug resistance has opened the possibility for the development of new drugs aimed at these targets. In this respect epigenetics and epigenomics are becoming important tools not only for therapeutic intervention [19, 23, 24, 26, 45], but also as predictor of drug response [46, 47]. In particular, histone demethylases have gained a particular importance because of their central role in a variety of aspects of the cells’ functionality and as therapeutic targets [23, 48].

In this report we show that targeting KDMs with synthetic inhibitors of their activity can interfere with tumor cell growth *in vitro* and *in vivo*. Having identified KDM5A as a demethylase involved in TMZ resistance in GB, we targeted this enzyme with JIB 04, a multi-KDM inhibitor with maximal activity against KDM5A, [12, 20–22, 24] and with CPI 455 a selective KDM5 inhibitor [24]. We showed that both molecules can efficiently inhibit GB cell growth in native and TMZ-resistant GB cells. JIB 04 is sensibly more potent than CPI 455 as antineoplastic agent and was utilized for all subsequent experiments. From a mechanistic point of view, we have shown that JIB 04 demethylates H3K4 and activates the expression of cancer-inhibiting genes, interferes with the PI3K/AKT pathway, one of the central survival mechanisms of cancer cells and that 60-minutes treatments are sufficient to activate death mechanisms and to drastically reduce or abolish the clonogenic properties of GB cells. The higher potency of JIB 04 over CPI 455 could be explained by its inhibitory activity over many KDMs possibly involved in GB cells proliferation and in drug resistance. Indeed we have previously shown that several KDMs inhibited by JIB 04 are overexpressed in GB and that their expression is transiently increased during the acquisition of TMZ resistance [12]. The determination of the precise role of these KDMs in GB is presently underway.

We have shown that JIB 04 synergizes with TMZ and can pass the BBB. In this respect our pilot *in vivo* study supports the concept that, in principle, this molecule can be the prototype of a novel class of drugs. It can be foreseen that the rapid advances on the knowledge of KDMs and KDMi(s) [23] will lead to the discovery of more selective and potent KDM inhibitors [24, 25] that will likely open the possibility to therapeutically finely tune histone methylation and that could be amenable for the utilization in a clinical setting

## SUPPLEMENTARY MATERIALS FIGURES AND TABLES



## References

[1] Ellis HP, Greenslade M, Powell B, Spiteri I, Sottoriva A, Kurian KM (2015). Current Challenges in Glioblastoma: Intratumour Heterogeneity, Residual Disease, and Models to Predict Disease Recurrence. Frontiers in oncology.

[2] Florio T, Barbieri F (2012). The status of the art of human malignant glioma management: the promising role of targeting tumor-initiating cells. Drug Discov Today.

[3] Friedman HS, Kerby T, Calvert H (2000). Temozolomide and treatment of malignant glioma. Clin Cancer Res.

[4] Stupp R, Mason WP, van den Bent MJ, Weller M, Fisher B, Taphoorn MJ, Belanger K, Brandes AA, Marosi C, Bogdahn U, Curschmann J, Janzer RC, Ludwin SK (2005). European Organisation for Research and Treatment of Cancer Brain Tumor and Radiotherapy Groups, and National Cancer Institute of Canada Clinical Trials Group. Radiotherapy plus concomitant and adjuvant temozolomide for glioblastoma. N Engl J Med.

[5] Hegi ME, Diserens AC, Gorlia T, Hamou MF, de Tribolet N, Weller M, Kros JM, Hainfellner JA, Mason W, Mariani L, Bromberg JE, Hau P, Mirimanoff RO (2005). MGMT gene silencing and benefit from temozolomide in glioblastoma. N Engl J Med.

[6] Dunn GP, Rinne ML, Wykosky J, Genovese G, Quayle SN, Dunn IF, Agarwalla PK, Chheda MG, Campos B, Wang A, Brennan C, Ligon KL, Furnari F (2012). Emerging insights into the molecular and cellular basis of glioblastoma. Genes Dev.

[7] Cahill DP, Codd PJ, Batchelor TT, Curry WT, Louis DN (2008). MSH6 inactivation and emergent temozolomide resistance in human glioblastomas. Clinical neurosurgery.

[8] Munoz JL, Rodriguez-Cruz V, Ramkissoon SH, Ligon KL, Greco SJ, Rameshwar P (2015). Temozolomide resistance in glioblastoma occurs by miRNA-9-targeted PTCH1, independent of sonic hedgehog level. Oncotarget.

[9] Oberstadt MC, Bien-Moller S, Weitmann K, Herzog S, Hentschel K, Rimmbach C, Vogelgesang S, Balz E, Fink M, Michael H, Zeden JP, Bruckmuller H, Werk AN (2013). Epigenetic modulation of the drug resistance genes MGMT, ABCB1 and ABCG2 in glioblastoma multiforme. BMC Cancer.

[10] Shinsato Y, Furukawa T, Yunoue S, Yonezawa H, Minami K, Nishizawa Y, Ikeda R, Kawahara K, Yamamoto M, Hirano H, Tokimura H, Arita K (2014). Reduction of MLH1 and PMS2 confers temozolomide resistance and is associated with recurrence of glioblastoma. Oncotarget.

[11] Kitange GJ, Mladek AC, Carlson BL, Schroeder MA, Pokorny JL, Cen L, Decker PA, Wu W, Lomberk GA, Gupta SK, Urrutia RA, Sarkaria JN (2012). Inhibition of histone deacetylation potentiates the evolution of acquired temozolomide resistance linked to MGMT upregulation in glioblastoma xenografts. Clin Cancer Res.

[12] Banelli B, Carra E, Barbieri F, Wurth R, Parodi F, Pattarozzi A, Carosio R, Forlani A, Allemanni G, Marubbi D, Florio T, Daga A, Romani M (2015). The histone demethylase KDM5A is a key factor for the resistance to temozolomide in glioblastoma. Cell Cycle.

[13] Liau BB, Sievers C, Donohue LK, Gillespie SM, Flavahan WA, Miller TE, Venteicher AS, Hebert CH, Carey CD, Rodig SJ, Shareef SJ, Najm FJ, van Galen P (2017). Adaptive Chromatin Remodeling Drives Glioblastoma Stem Cell Plasticity and Drug Tolerance. Cell Stem Cell.

[14] Sareddy GR, Nair BC, Krishnan SK, Gonugunta VK, Zhang QG, Suzuki T, Miyata N, Brenner AJ, Brann DW, Vadlamudi RK (2013). KDM1 is a novel therapeutic target for the treatment of gliomas. Oncotarget.

[15] Singh MM, Manton CA, Bhat KP, Tsai WW, Aldape K, Barton MC, Chandra J (2011). Inhibition of LSD1 sensitizes glioblastoma cells to histone deacetylase inhibitors. Neuro Oncol.

[16] Kozono D, Li J, Nitta M, Sampetrean O, Gonda D, Kushwaha DS, Merzon D, Ramakrishnan V, Zhu S, Zhu K, Matsui H, Harismendy O, Hua W (2015). Dynamic epigenetic regulation of glioblastoma tumorigenicity through LSD1 modulation of MYC expression. Proc Natl Acad Sci USA.

[17] Grasso CS, Tang Y, Truffaux N, Berlow NE, Liu L, Debily MA, Quist MJ, Davis LE, Huang EC, Woo PJ, Ponnuswami A, Chen S, Johung TB (2015). Functionally defined therapeutic targets in diffuse intrinsic pontine glioma. Nature medicine.

[18] Hashizume R, Andor N, Ihara Y, Lerner R, Gan H, Chen X, Fang D, Huang X, Tom MW, Ngo V, Solomon D, Mueller S, Paris PL (2014). Pharmacologic inhibition of histone demethylation as a therapy for pediatric brainstem glioma. Nature medicine.

[19] Natoli G, Testa G, De Santa F (2009). The future therapeutic potential of histone demethylases: A critical analysis. Curr Opin Drug Discov Devel.

[20] Sharma SV, Lee DY, Li B, Quinlan MP, Takahashi F, Maheswaran S, McDermott U, Azizian N, Zou L, Fischbach MA, Wong KK, Brandstetter K, Wittner B (2010). A chromatin-mediated reversible drug-tolerant state in cancer cell subpopulations. Cell.

[21] Hou J, Wu J, Dombkowski A, Zhang K, Holowatyj A, Boerner JL, Yang ZQ (2012). Genomic amplification and a role in drug-resistance for the KDM5A histone demethylase in breast cancer. Am J Transl Res.

[22] Yan H, Chen X, Zhang Q, Qin J, Li H, Liu C, Calhoun-Davis T, Coletta LD, Klostergaard J, Fokt I, Skora S, Priebe W, Bi Y (2011). Drug-tolerant cancer cells show reduced tumor-initiating capacity: depletion of CD44 cells and evidence for epigenetic mechanisms. PLoS One.

[23] McAllister TE, England KS, Hopkinson RJ, Brennan PE, Kawamura A, Schofield CJ (2016). Recent Progress in Histone Demethylase Inhibitors. J Med Chem.

[24] Vinogradova M, Gehling VS, Gustafson A, Arora S, Tindell CA, Wilson C, Williamson KE, Guler GD, Gangurde P, Manieri W, Busby J, Flynn EM, Lan F (2016). An inhibitor of KDM5 demethylases reduces survival of drug-tolerant cancer cells. Nature chemical biology.

[25] Gale M, Sayegh J, Cao J, Norcia M, Gareiss P, Hoyer D, Merkel JS, Yan Q (2016). Screen-identified selective inhibitor of lysine demethylase 5A blocks cancer cell growth and drug resistance. Oncotarget.

[26] Wang L, Chang J, Varghese D, Dellinger M, Kumar S, Best AM, Ruiz J, Bruick R, Pena-Llopis S, Xu J, Babinski DJ, Frantz DE, Brekken RA (2013). A small molecule modulates Jumonji histone demethylase activity and selectively inhibits cancer growth. Nat Commun.

[27] Biddlestone-Thorpe L, Sajjad M, Rosenberg E, Beckta JM, Valerie NC, Tokarz M, Adams BR, Wagner AF, Khalil A, Gilfor D, Golding SE, Deb S, Temesi DG (2013). ATM kinase inhibition preferentially sensitizes p53-mutant glioma to ionizing radiation. Clin Cancer Res.

[28] Shi W, Palmer JD, Werner-Wasik M, Andrews DW, Evans JJ, Glass J, Kim L, Bar-Ad V, Judy K, Farrell C, Simone N, Liu H, Dicker AP Phase I trial of panobinostat and fractionated stereotactic re-irradiation therapy for recurrent high grade gliomas. J Neurooncol.

[29] Stepp MW, Folz RJ, Yu J, Zelko IN (2014). The c10orf10 gene product is a new link between oxidative stress and autophagy. Biochimica et biophysica acta.

[30] Shoshani T, Faerman A, Mett I, Zelin E, Tenne T, Gorodin S, Moshel Y, Elbaz S, Budanov A, Chajut A, Kalinski H, Kamer I, Rozen A (2002). Identification of a novel hypoxia-inducible factor 1-responsive gene, RTP801, involved in apoptosis. Mol Cell Biol.

[31] Schaaf MB, Keulers TG, Vooijs MA, Rouschop KM (2016). LC3/GABARAP family proteins: autophagy-(un)related functions. FASEB J.

[32] Massacesi C, Di Tomaso E, Urban P, Germa C, Quadt C, Trandafir L, Aimone P, Fretault N, Dharan B, Tavorath R, Hirawat S (2016). PI3K inhibitors as new cancer therapeutics: implications for clinical trial design. OncoTargets and therapy.

[33] Chakravarti A, Zhai G, Suzuki Y, Sarkesh S, Black PM, Muzikansky A, Loeffler JS (2004). The prognostic significance of phosphatidylinositol 3-kinase pathway activation in human gliomas. J Clin Oncol.

[34] Carmo A, Carvalheiro H, Crespo I, Nunes I, Lopes MC (2011). Effect of temozolomide on the U-118 glioma cell line. Oncology letters.

[35] Li X, Wu C, Chen N, Gu H, Yen A, Cao L, Wang E, Wang L (2016). PI3K/Akt/mTOR signaling pathway and targeted therapy for glioblastoma. Oncotarget.

[36] Johnson BE, Mazor T, Hong C, Barnes M, Aihara K, McLean CY, Fouse SD, Yamamoto S, Ueda H, Tatsuno K, Asthana S, Jalbert LE, Nelson SJ (2014). Mutational analysis reveals the origin and therapy-driven evolution of recurrent glioma. Science.

[37] Chou TC (2010). Drug combination studies and their synergy quantification using the Chou-Talalay method. Cancer Res.

[38] Seystahl K, Wick W, Weller M Therapeutic options in recurrent glioblastoma-An update. Crit Rev Oncol Hematol.

[39] Frosina G (2015). The glioblastoma problem: targeting by combined medicinal chemistry approaches. Current medicinal chemistry.

[40] Yamanaka R, Hayano A (2014). Experiences and expectations for glioma immunotherapeutic approaches. Front Oncol.

[41] Honeychurch J, Cheadle EJ, Dovedi SJ, Illidge TM (2015). Immuno-regulatory antibodies for the treatment of cancer. Expert Opin Biol Ther.

[42] Kim JE, Patel MA, Mangraviti A, Kim ES, Theodros D, Velarde E, Liu A, Sankey E, Tam A, Xu H, Mathios D, Jackson CM, Harris-Bookman S (2017). Combination therapy with anti-PD-1, anti-TIM-3, and focal radiation results in regression of murine gliomas.

[43] Lim M, Weller M, Chiocca EA (2016). Current State of Immune-Based Therapies for Glioblastoma. Am Soc Clin Oncol Educ Book.

[44] Bouffet E, Larouche V, Campbell BB, Merico D, de Borja R, Aronson M, Durno C, Krueger J, Cabric V, Ramaswamy V, Zhukova N, Mason G, Farah R (2016). Immune Checkpoint Inhibition for Hypermutant Glioblastoma Multiforme Resulting From Germline Biallelic Mismatch Repair Deficiency. J Clin Oncol.

[45] Wang J, Lu F, Ren Q, Sun H, Xu Z, Lan R, Liu Y, Ward D, Quan J, Ye T, Zhang H (2011). Novel histone demethylase LSD1 inhibitors selectively target cancer cells with pluripotent stem cell properties. Cancer Res.

[46] Baer-Dubowska W, Majchrzak-Celinska A, Cichocki M (2011). Pharmocoepigenetics: a new approach to predicting individual drug responses and targeting new drugs. Pharmacol Rep.

[47] Ivanov M, Kacevska M, Ingelman-Sundberg M (2012). Epigenomics and interindividual differences in drug response. Clin Pharmacol Ther.

[48] Rotili D, Mai A (2011). Targeting Histone Demethylases: A New Avenue for the Fight against Cancer. GenesCancer.

[49] Gangemi RM, Griffero F, Marubbi D, Perera M, Capra MC, Malatesta P, Ravetti GL, Zona GL, Daga A, Corte G (2009). SOX2 silencing in glioblastoma tumor-initiating cells causes stop of proliferation and loss of tumorigenicity. Stem Cells.

[50] Griffero F, Daga A, Marubbi D, Capra MC, Melotti A, Pattarozzi A, Gatti M, Bajetto A, Porcile C, Barbieri F, Favoni RE, Lo Casto M, Zona G (2009). Different response of human glioma tumor-initiating cells to epidermal growth factor receptor kinase inhibitors. J Biol Chem.

