# Supplementary material for: Small molecules targeting histone demethylase genes (KDMs) inhibit growth of temozolomide-resistant glioblastoma cells
Source: Oncotarget. 2017 Apr 4;8(21):34896–910. doi: 10.18632/oncotarget.16820 (PMC5471020; doi:10.18632/oncotarget.16820)
Supplement: Supplementary file 1 [file oncotarget-08-34896-s001.pdf]

Small molecules targeting histone demethylase genes (KDMs) inhibit growth of Temozolomide-resistant glioblastoma cells

Supplementary Materials

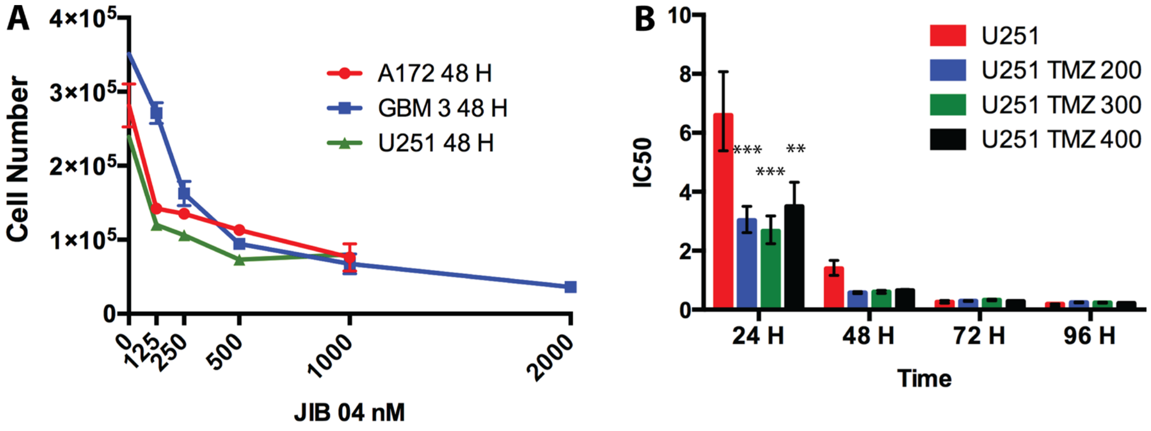

C

IC 50 (µM) values and Confidence Intervals (CI) of TMZ-Resistant and native U251 cells exposed to TMZ and JIB 04

	TMZ	JIB 04			
	96 H	24 H	48 H	72 H	96 H
U251	183.8 (CI 145.1-232.7)	6.584 (CI 5.38-8.06)	1.386 (CI 1.16-1.66)	0.255 (CI 0.22-0.31)	0.127 (CI 0.11-0.15)
U251 TMZ 200	525.3 (CI 449.6-613.8)	3.027 (CI 2.64-3.51)	0.575 (CI 0.54-0.61)	0.296 (CI 0.28-0.31)	0.246 (CI 0.23-0.26)
U251 TMZ 300	752.8 (CI 620.3-913.6)	2.669 (CI 2.24-3.18)	0.603 (CI 0.55-0.65)	0.327 (CI 0.30- 0.35)	0.240 (CI 0.22-0.26)
U251 TMZ 400	877.1 (CI 792.5-970.7)	4.405 (CI 3.81-5.32)	0.651 (CI 0.62-0.68)	0.287 (CI 0.27.0.31)	0.221 (CI 0.21-0.23)

Supplementary Figure 1: (Panel **A**) Effect of JIB 04 on cell proliferation. Cell were incubated in the presence of JIB 04 for 72 hours. The number of viable cells was measured with the Cell Count and Viability Assay utilizing a MUSE Cell Analyzer (Millipore). The significance of the difference between treated and control cells is reported in Supplementary Table 1C. (Panel **B**) plot of IC50 values of U251 cells native and resistant to 200, 300 and 400 µM TMZ treated with JIB 04 for 24, 48, 72 and 96 hours. The difference of the IC50 values is significantly lower in the TMZ resistant cell lines at 24 hours. At 48 hours, although the difference is not significant, the IC50 of native cells is twice that of the TMZ-resistant derivatives. The significance of the differences was calculated by ANOVA with the Bonferroni's correction for multiple comparisons and is indicated for each point (*P = 0.05; **P = 0.01; ***P = 0.001). (Panel **C**) the numerical values utilized for the plot of Panel B.

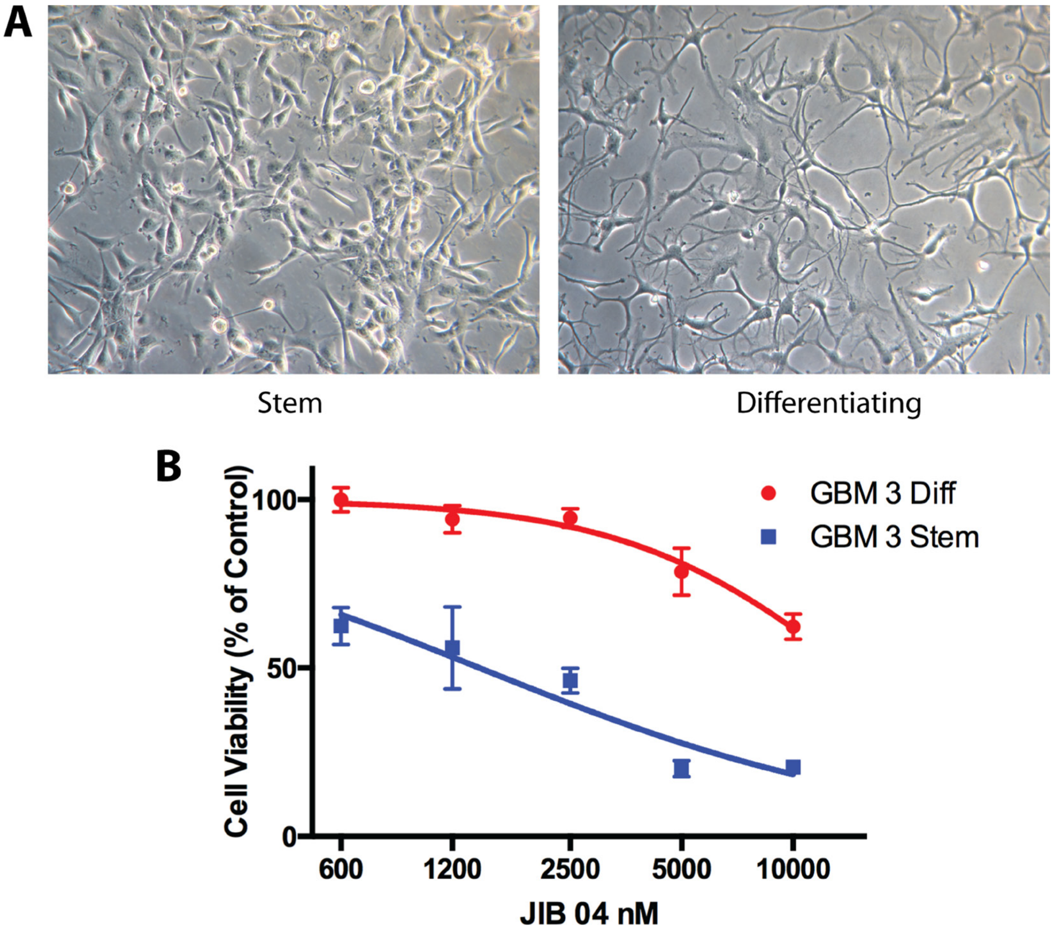

Supplementary Figure 2: Activity of JIB 04 on GB primary cultures grown under stem or differentiating conditions. (Panel **A**) morphology of GBM3 cells grown in stem-permissive medium and undergoing differentiation when grown in a serum-containing medium. (Panel **B**) MTS assay on stem and differentiating GBM3 cells treated with JIB 04.

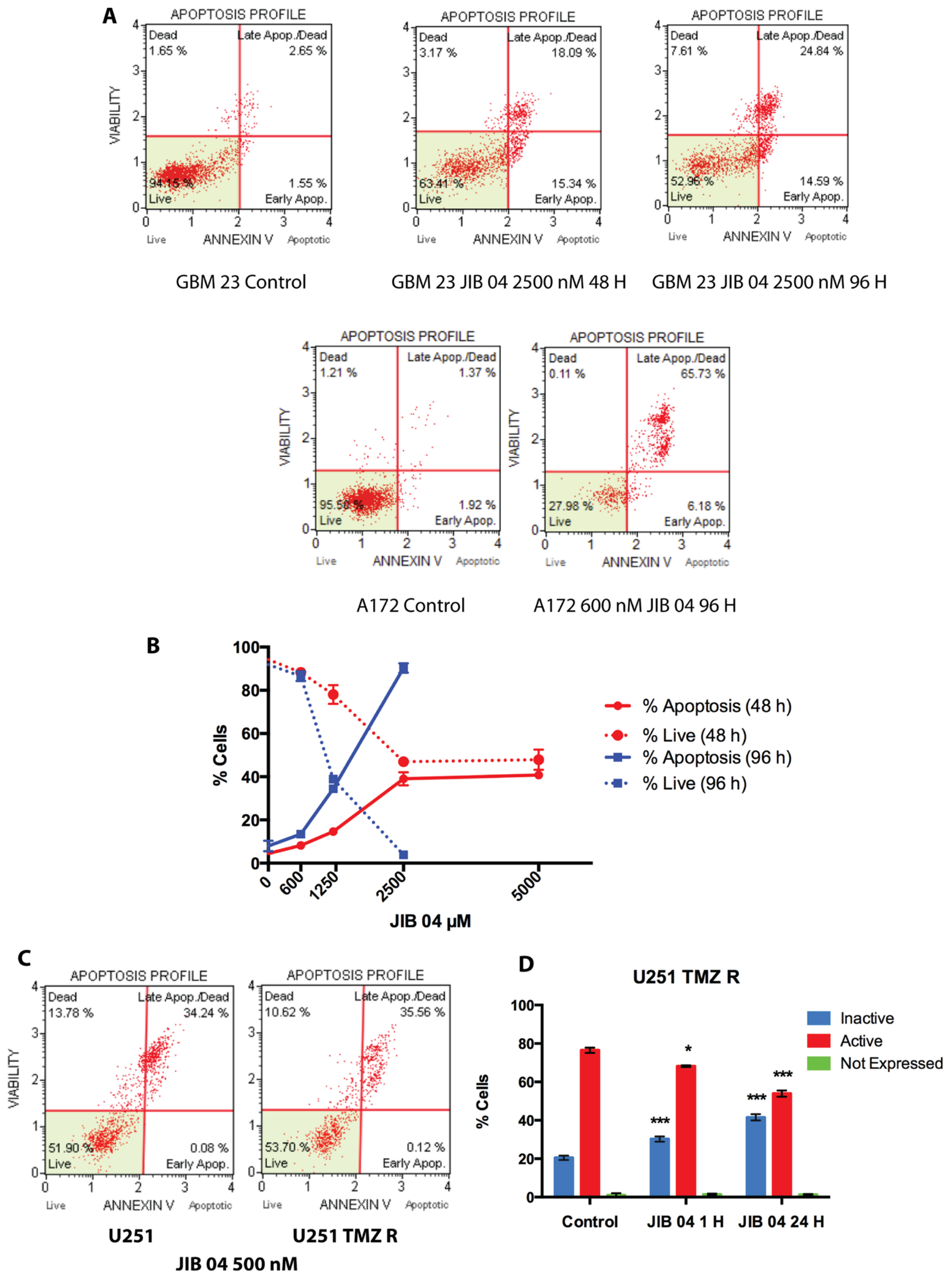

Supplementary Figure 3: (Panel **A**) Annexin V staining of the stem-enriched primary GB culture GBM 23 and of the A172 GB cell line treated with JIB 04 for 96 hours. Annexin V staining was evaluated with the Muse Annexin V and Cell Death kit utilizing a MUSE cell analyzer (Millipore. Milano, Italy). (Panel **B**) total apoptotic and live cells after treatment of GBM 3 cells for 48 and 96 hours with different concentrations of JIB 04. Annexin V staining was evaluated with the Muse Annexin V and Cell Death kit utilizing a MUSE cell analyzer (Millipore. Milano, Italy). The statistical evaluation of the effect of JIB 04 on GBM3 cells is reported in Supplementary Table 2. (Panel **C**) Scatterograms showing the induction of apoptosis by JIB 04, evaluated by Annexin V staining, of native and TMZ resistant U251 cells treated for 96 hours. (Panel **D**) Flow-cytometry analysis of Akt phosphorylation after 1 and 24 hours of treatment with JIB04 of U251 TMZ-resistant cells. These plots show the presence of significant decrease in the phosphorylation of Akt indicating inactivation of the PI3K pathway. Each datapoint represents the mean value of two experiments. The significance of the differences respect the untreated cells was calculated determined by ANOVA with the Bonferroni's correction for multiple comparisons and is indicated for each point (**P* = 0.05; ***P* = 0.01; ****P* = 0.001).

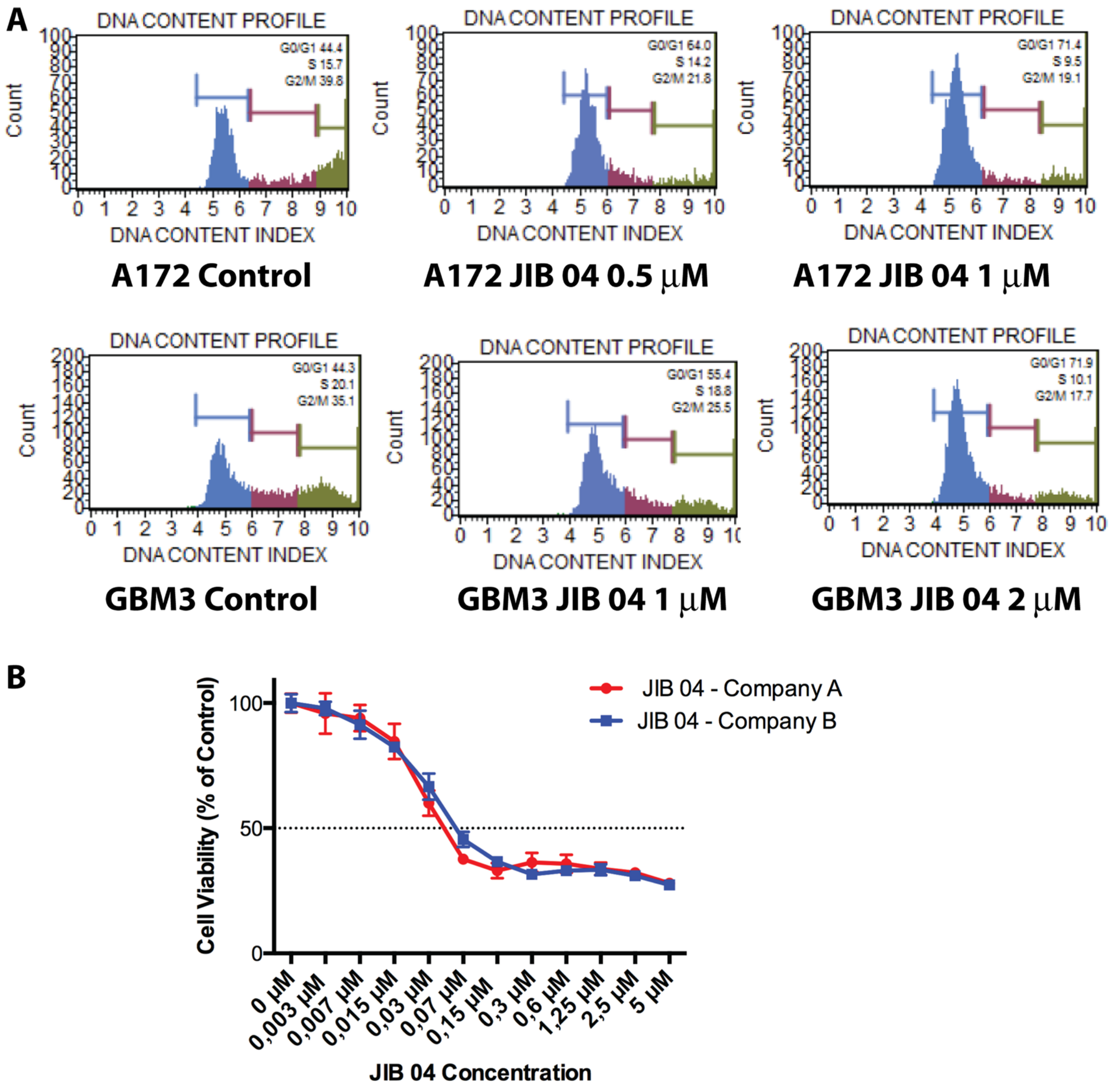

Supplementary Figure 4: (Panel **A**) Cell cycle analysis of A172 and GBM3 cells exposed to JIB 04 for 48 hours showing the progressive, dose-dependent accumulation of the cells in G0/G1. (Panel **B**) MTS analysis of A172 cells showing identical effect after treatment with JIB 04 from two different Companies.

A

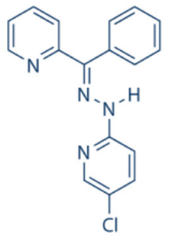

($C_{17}H_{13}ClN_4$)

B

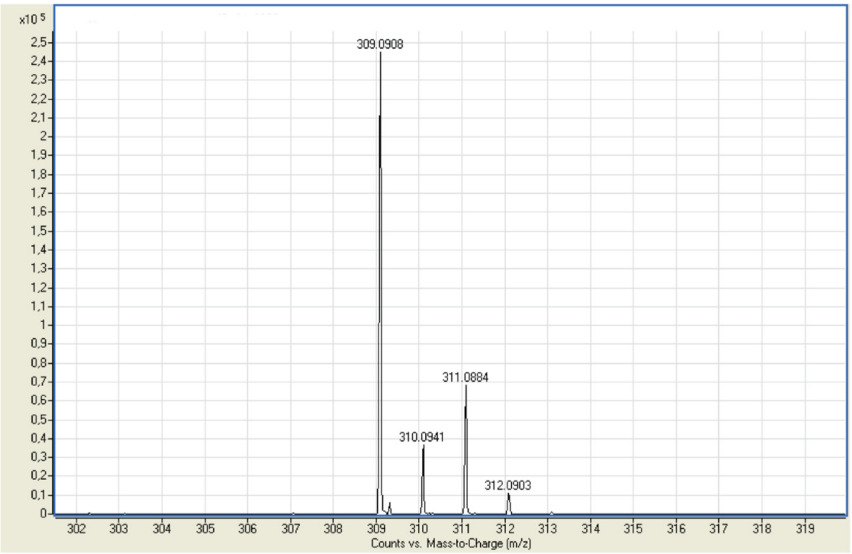

C

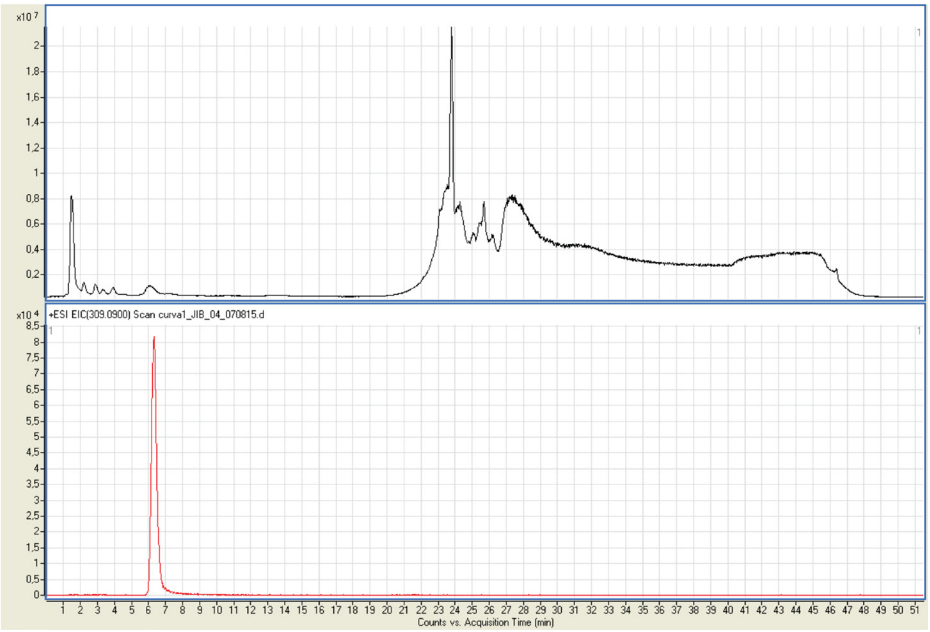

Supplementary Figure 5: Mass spectrometry analysis for the identification of JIB 04 in biological samples. (Panel **A**) Chemical structure of JIB-04. (Panel **B**) MS analysis in positive polarity: Isotopic pattern of JIB-04. (Panel **C**) HPLC/MS analysis of JIB-04 standard: Total ion current (upper panel) and extracted ion current peak area of m/z 309.09 $[M+H]^+$ (lower panel).

Supplementary Table 1: Statistical evaluation of JIB 04 and TMZ sensitivity at different molecule concentrations

(A) Cell viability (Figure 1, Panels A and B) (Effect of TMZ treatment compared to untreated cells)

	TMZ µM						
**Cell Line**	**25**	**50**	**100**	**200**	**400**	**800**	**1600**
A172	NS	NS	NS	NS	***	***	***
CAS1	NS	NS	NS	NS	**	***	***
DBTRG	***	***	***	***	***	***	***
U251	*	**	NS	***	***	***	***
U87 MG	NS	NS	*	***	***	***	***
GBM 3	**	**	*	***	***	***	***
GBM23	NS	NS	*	**	***	***	***

(B) Cell viability (Figure 1, Panels C and D) (Effect of JIB 04 treatment compared to untreated cells)

	JIB 04 nM						
**Cell Line**	**75**	**150**	**300**	**600**	**1250**	**2500**	**5000**
A172	NS	NS	***	***	***	***	***
CAS1	NS	NS	**	***	***	***	***
DBTRG	NS	***	***	***	***	***	***
U251	NS	**	***	***	***	***	***
U87 MG	NS	NS	NS	NS	***	***	***
	JIB 04 nM						
	150	300	600	1250	2500	5000	10000
GBM 3	NS	NS	NS	NS	***	***	***
GBM 23	NS	NS	NS	*	***	***	***

(C) Cell proliferation (Supplementary Figure 1, panel A) (Effect of TMZ treatment compared to untreated cells)

	JIB 04 nM				
**Cell Line**	**125**	**250**	**500**	**1000**	**2000**
A172	***	***	***	***	ND
U251	NS	**	***	***	ND
GBM 3	**	***	***	***	***

Two-way ANOVA. Significance (Bonferroni post hoc) of each data point compared to the untreated control.
NS: not significant.
ND: not done.
*$P < 0.05$.
**$P < 0.01$.
***$P < 0.001$.

Supplementary Table 2: Statistical evaluation of apoptosis and cell survival after treatment with JIB 04 at different molecule concentrations and time in GBM 3 cells (Supplementary Figure 3, Panel B)

(A) Apoptosis

	JIB 04 nM				
**Incubation time**	**0**	**600**	**1250**	**2500**	**5000**
48 h	-	NS	***	***	***
96 h	-	*	***	***	***
48 h vs 96 h	-	NS	***	***	***

(B) Cell Survival

	JIB 04 nM				
**Incubation time**	**0**	**600**	**1250**	**2500**	**5000**
48 h	-	NS	***	***	***
96 h	-	NS	**	***	***
48 h vs 96 h	-	NS	***	***	***

Two-way ANOVA. Significance (Bonferroni post hoc) of each data point compared to the untreated control.
NS: not significant.
*P < 0.05.
**P < 0.01.
***P < 0.001.

Supplementary Table 3: Statistical evaluation of JIB 04 sensitivity at different treatment time

(Figure 4, Panel A)

	JIB 04 nM						
**Treatment Time**	**150**	**300**	**600**	**1200**	**2500**	**5000**	**10000**
1 H	NS	NS	NS	*	**	***	***
4 H	NS	NS	NS	NS	**	***	***
24 H	NS	NS	NS	**	***	***	***
48 H	NS	*	***	***	***	***	***
72 H	NS	*	***	***	***	***	***

One-way ANOVA. Significance (Bonferroni post hoc) of each data point compared to the untreated control.
NS: not significant.
*P < 0.05.
**P < 0.01.
***P < 0.001.
